# Supplementary material for: Variational Bayesian Parameter Estimation Techniques for the General Linear Model
Source: Front Neurosci. 2017 Sep 15;11:504. doi: 10.3389/fnins.2017.00504 (PMC5605759; doi:10.3389/fnins.2017.00504)
Supplement: Supplementary file 1 [file DataSheet1.pdf]

Supplementary Material

Contents

S1 Free energy algorithm derivations **1**
S1.1 Preliminaries 1
S1.2 Variational Bayes 3
S1.3 Variational maximum likelihood 8
S1.4 Restricted maximum likelihood 10
S1.5 Maximum likelihood 13

S2 Foundations of variational Bayes **14**
S2.1 Preliminaries 14
S2.2 Entropies of distributions of random vectors 18
S2.3 Probability-theoretic variational Bayes 19

S3 Cumulative averages without outlier removal **24**

S4 SPM12 ReML estimation **25**

S5 Model recovery free energy contributions **26**

S1 Free energy algorithm derivations

In this section, we evaluate the VB, VML, ReML, and ML free energies for the GLM and derive update equations for their maximization. The notation follows the applied approach used in the main text. We commence with some remarks on additional notation and matrix differentation.

S1.1 Preliminaries

Expectations

To ease the notation, we will often write the expectation of a function f of random variable x under the probability distribution $p(x)$ using the expectation operator

$$\langle f(x)\rangle_{p(x)} = \int f(x)p(x)\,dx \tag{S1.1}$$

Furthermore, on numerous occasions, we require the following property of expectations of multivariate random variables $x \in \mathbb{R}^d$ under normal distributions: for $x, m, \mu \in \mathbb{R}^d, \Sigma \in \mathbb{R}^{d \times d}$ p.d. and $A \in \mathbb{R}^{d \times d}$ it holds that

$$\langle (x-m)^T A (x-m) \rangle_{N(x;\mu,\Sigma)} = (\mu - m)^T A (\mu - m) + \mathrm{tr}(A\Sigma) \tag{S1.2}$$

(see e.g. Petersen and Pedersen (2012), eq. (380)).

Gradient and Hessian

The gradient and Hessian of a real-valued function

$$f : \mathbb{R}^n \to \mathbb{R}, x \mapsto f(x) \tag{S1.3}$$

evaluated at a point $a \in \mathbb{R}^n$ will be denoted by

$$\nabla f(a) := \left(\frac{\partial}{\partial x_1} f(a), ..., \frac{\partial}{\partial x_n} f(a) \right)^T \in \mathbb{R}^n \tag{S1.4}$$

and

$$H_f(a) := \begin{pmatrix} \frac{\partial^2}{\partial x_1^2} f(a) & \cdots & \frac{\partial^2}{\partial x_1 \partial x_n} f(a) \\ \vdots & \ddots & \vdots \\ \frac{\partial^2}{\partial x_n \partial x_1} f(a) & \cdots & \frac{\partial^2}{\partial x_n^2} f(a) \end{pmatrix} \in \mathbb{R}^{n \times n}. \tag{S1.5}$$

When it eases the notation, we also occasionally denote the partial derivative of f with respect to x_i evaluated at $a \in \mathbb{R}^n$ by $\frac{\partial}{\partial x_i} f|_{x=a}$.

Matrix differentiation

The following matrix differentiation rules are used in the subsequent derivations (Petersen and Pedersen, 2012). For a matrix A depending on a scalar parameter x, we have

$$\frac{\partial |A|}{\partial x} = |A| \,\mathrm{tr} \left(A^{-1} \frac{\partial A}{\partial x} \right) \tag{S1.6}$$

$$\frac{\partial \ln |A|}{\partial x} = \mathrm{tr} \left(A^{-1} \frac{\partial A}{\partial x} \right) \tag{S1.7}$$

$$\frac{\partial A^{-1}}{\partial x} = -A^{-1} \frac{\partial A}{\partial x} A^{-1} \tag{S1.8}$$

$$\frac{\partial \,\mathrm{tr}\,(A)}{\partial x} = \mathrm{tr} \left(\frac{\partial A}{\partial x} \right). \tag{S1.9}$$

For a matrix A depending on a two-dimensional vector $x = (x_1, x_2)$, the second-order partial derivatives of its inverse are

$$\frac{\partial^2 A^{-1}}{\partial x_1^2} = 2A^{-1} \frac{\partial A}{\partial x_1} A^{-1} \frac{\partial A}{\partial x_1} A^{-1} - A^{-1} \frac{\partial^2 A}{\partial x_1^2} A^{-1} \tag{S1.10}$$

$$\frac{\partial^2 A^{-1}}{\partial x_2^2} = 2A^{-1} \frac{\partial A}{\partial x_2} A^{-1} \frac{\partial A}{\partial x_2} A^{-1} - A^{-1} \frac{\partial^2 A}{\partial x_2^2} A^{-1} \tag{S1.11}$$

and

$$\frac{\partial^2 A^{-1}}{\partial x_1 \partial x_2} = \frac{\partial^2 A^{-1}}{\partial x_2 \partial x_1} = A^{-1}\frac{\partial A}{\partial x_1}A^{-1}\frac{\partial A}{\partial x_2}A^{-1} + A^{-1}\frac{\partial A}{\partial x_2}A^{-2}\frac{\partial A}{\partial x_1}A^{-1} - A^{-1}\frac{\partial^2 A}{\partial x_1 \partial x_2}A^{-1} \quad \text{(S1.12)}$$

assuming that A has continuous second derivatives, such that the symmetry of second-order derivatives (Schwarz's theorem) holds. For the update equations of the matrix parameters S_β and S_λ, we also need to compute derivatives regarding matrices. We have

$$\frac{\partial \ln(|A|)}{\partial A} = A^{-1} \quad \text{(S1.13)}$$

and for matrices A and B of matching dimensions

$$\frac{\partial \operatorname{tr}(AB)}{\partial A} = B^T. \quad \text{(S1.14)}$$

S1.2 Variational Bayes

Evaluation of the VB free energy

To evaluate the VB free energy, we first rewrite it from its definition in eq. (20) in the main text as follows

$$\begin{aligned} F^{VB}(q(\beta)q(\lambda)) &= \langle \ln\left(\frac{p(y,\beta,\lambda)}{q(\beta)q(\lambda)}\right)\rangle_{q(\beta)q(\lambda)} \\ &= \langle \ln p(y|\beta,\lambda)\rangle_{q(\beta)q(\lambda)} + \langle \ln p(\beta)\rangle_{q(\beta)} + \langle \ln p(\lambda)\rangle_{q(\lambda)} \\ &\quad - \langle q(\beta)\rangle_{q(\beta)} - \langle q(\lambda)\rangle_{q(\lambda)}. \end{aligned} \quad \text{(S1.15)}$$

Using (S1.2), the second and third term on the right-hand side of (S1.15) can be evaluated exactly, yielding

$$\langle \ln p(\beta)\rangle_{q(\beta)} = -\frac{p}{2}\ln 2\pi - \frac{1}{2}\ln|\Sigma_\beta| - \frac{1}{2}(m_\beta - \mu_\beta)^T\Sigma_\beta^{-1}(m_\beta - \mu_\beta) - \frac{1}{2}\operatorname{tr}(\Sigma_\beta^{-1}S_\beta) \quad \text{(S1.16)}$$

and

$$\langle \ln p(\lambda)\rangle_{q(\lambda)} = -\frac{k}{2}\ln 2\pi - \frac{1}{2}\ln|\Sigma_\lambda| - \frac{1}{2}(m_\lambda - \mu_\lambda)^T\Sigma_\lambda^{-1}(m_\lambda - \mu_\lambda) - \frac{1}{2}\operatorname{tr}(\Sigma_\lambda^{-1}S_\lambda). \quad \text{(S1.17)}$$

corresponding to terms 6 - 13 of eq. (28) in the main text. The fourth and the fifth term on the right-hand side of (S1.15) correspond to the entropies of the variational distributions, which given their Gaussian form are given as function of their respective covariance matrices (e.g., Bishop, 2006)

$$H(q(\beta)) = -\langle \ln q(\beta)\rangle_{q(\beta)} = \frac{p}{2}\ln(2\pi e) + \frac{1}{2}\ln|S_\beta|, \quad \text{(S1.18)}$$

$$H(q(\lambda)) = -\langle \ln q(\lambda)\rangle_{q(\lambda)} = \frac{k}{2}\ln(2\pi e) + \frac{1}{2}\ln|S_\lambda|. \quad \text{(S1.19)}$$

Eqs. (S1.18) and (S1.19) correspond to terms 14 to 16 of eq. (28) in the main text.

Finally, we consider the first term of (S1.15). Based on the definition of $p(y|\beta,\lambda)$, the expectation with respect to $q(\beta)$ can be evaluated exactly, yielding

$$\begin{aligned}\langle \ln p(y|\beta,\lambda)\rangle_{q(\beta)q(\lambda)} = &-\frac{n}{2}\ln 2\pi - \frac{1}{2}\langle \ln|V_\lambda|\rangle_{q(\lambda)} \\ &- \frac{1}{2}\langle (y-Xm_\beta)^T V_\lambda^{-1}(y-Xm_\beta)\rangle_{q(\lambda)} \\ &- \frac{1}{2}\langle \mathrm{tr}(V_\lambda^{-1}XS_\beta X^T)\rangle_{q(\lambda)}.\end{aligned} \tag{S1.20}$$

To make it possible to evaluate the remaining expectations, we use a second order Taylor approximation. Let

$$f:\mathbb{R}^k \to \mathbb{R}, \lambda \mapsto f(\lambda) \tag{S1.21}$$

denote a real-valued function of λ. Then

$$f(\lambda) \approx f(m_\lambda) + (\lambda - m_\lambda)^T \nabla f(m_\lambda) + \frac{1}{2}(\lambda - m_\lambda)^T H_f(m_\lambda)(\lambda - m_\lambda) \tag{S1.22}$$

in the vicinity of m_λ. If $q(\lambda)$ is sufficiently narrow, that is, if most of its mass is concentrated close to m_λ, we can thus approximate

$$\begin{aligned}\langle f(\lambda)\rangle_{q(\lambda)} &\approx f(m_\lambda) + \langle (\lambda - m_\lambda)^T \nabla f(m_\lambda)\rangle_{q(\lambda)} + \frac{1}{2}\langle (\lambda - m_\lambda)^T H_f(m_\lambda)(\lambda - m_\lambda)\rangle_{q(\lambda)} \\ &= f(m_\lambda) + \frac{1}{2}\mathrm{tr}\left(H_f(m_\lambda)S_\lambda\right).\end{aligned} \tag{S1.23}$$

This approximation needs to be applied to all expectations in equation (S1.20). Thus, using the linearity of the trace to subsume all Hessian matrices into

$$\begin{aligned}B_{m_\beta,S_\beta,m_\lambda} = &H_{\ln|V_\lambda|}(m_\lambda) + H_{(y-Xm_\beta)^T V_\lambda^{-1}(y-Xm_\beta)}(m_\lambda) \\ &+ H_{\mathrm{tr}(V_\lambda^{-1}XS_\beta X^T)}(m_\lambda),\end{aligned} \tag{S1.24}$$

thereby pooling the second-order terms, we arrive at terms 1 - 5 of equation (28) in the main text, and the derivation is complete.

Evaluation of $B_{m_\beta,S_\beta,m_\lambda}$

To estimate the VB free energy in practice, the Hessian matrices on the right-hand side of (S1.24) have to be evaluated. For the linear form of the error covariance matrix

$$V_\lambda := \exp(\lambda_1)I_n + \exp(\lambda_2)Q_2 \tag{S1.25}$$

the three Hessian matrices of (S1.24) can be evaluated analytically:

- $H_{\ln|V_\lambda|}$

Using (S1.7), the first order partial derivatives are given by

$$\frac{\partial \ln|V_\lambda|}{\partial \lambda_1} = \exp(\lambda_1)\,\mathrm{tr}\left(V_\lambda^{-1}\right) \tag{S1.26}$$

and

$$\frac{\partial \ln |V_\lambda|}{\partial \lambda_2} = \exp(\lambda_2) \operatorname{tr}\left(V_\lambda^{-1} Q_2\right). \tag{S1.27}$$

Exploiting the linearity of the trace operator (S1.9) and using (S1.8) for the derivative of the inverse yields the second order partial derivatives:

$$\begin{aligned}\frac{\partial^2 \ln |V_\lambda|}{\partial \lambda_1^2} &= \exp(\lambda_1) \operatorname{tr}\left(V_\lambda^{-1}\right) - \exp(2\lambda_1) \operatorname{tr}\left(V_\lambda^{-2}\right) \\ &= \exp(\lambda_1) \operatorname{tr}\left(V_\lambda^{-1}\right) - \big(\exp(\lambda_1) \operatorname{tr}\left(V_\lambda^{-2} V_\lambda\right) \\ &\qquad - \exp(\lambda_1+\lambda_2) \operatorname{tr}\left(V_\lambda^{-2} Q_2\right)\big) \\ &= \exp(\lambda_1+\lambda_2) \operatorname{tr}\left(V_\lambda^{-2} Q_2\right) ,\end{aligned} \tag{S1.28}$$

$$\begin{aligned}\frac{\partial^2 \ln |V_\lambda|}{\partial \lambda_2^2} &= \exp(\lambda_2) \operatorname{tr}\left(V_\lambda^{-1} Q_2\right) - \exp(2\lambda_2) \operatorname{tr}\left(V_\lambda^{-1} Q_2 V_\lambda^{-1} Q_2\right) \\ &= \exp(\lambda_2) \operatorname{tr}\left(V_\lambda^{-1} Q_2\right) - \big(\exp(\lambda_2) \operatorname{tr}\left(V_\lambda^{-1} V_\lambda V_\lambda^{-1} Q_2\right) \\ &\qquad - \exp(\lambda_1+\lambda_2) \operatorname{tr}\left(V_\lambda^{-2} Q_2\right)\big) \\ &= \exp(\lambda_1+\lambda_2) \operatorname{tr}\left(V_\lambda^{-2} Q_2\right),\end{aligned} \tag{S1.29}$$

and

$$\frac{\partial^2 \ln |V_\lambda|}{\partial \lambda_1 \partial \lambda_2} = \frac{\partial^2 \ln |V_\lambda|}{\partial \lambda_2 \partial \lambda_1} = -\exp(\lambda_1+\lambda_2) \operatorname{tr}\left(V_\lambda^{-2} Q_2\right), \tag{S1.30}$$

where in the last equation we used that the trace is invariant under cyclic permutations, e.g. $\operatorname{tr}(ABC) = \operatorname{tr}(CAB) = \operatorname{tr}(BCA)$.

- $H_{(y-Xm_\beta)^T V_\lambda^{-1}(y-Xm_\beta)}$

The Hessian matrix of $(y-Xm_\beta)^T V_\lambda^{-1}(y-Xm_\beta)$ only depends on the second order partial derivatives of the inverse of V_λ

$$\frac{\partial^2}{\partial \lambda_i \partial \lambda_j}\left((y-Xm_\beta)^T V_\lambda^{-1}(y-Xm_\beta)\right) = (y-Xm_\beta)^T \frac{\partial^2 V_\lambda^{-1}}{\partial \lambda_i \partial \lambda_j}(y-Xm_\beta) \tag{S1.31}$$

for $i,j \in \{1,2\}$. Applying (S1.10) to (S1.12) yields

$$\frac{\partial^2 V_\lambda^{-1}}{\partial \lambda_1^2} = \exp(\lambda_1) V_\lambda^{-2} - 2\exp(2\lambda_1) V_\lambda^{-3}, \tag{S1.32}$$

$$\frac{\partial^2 V_\lambda^{-1}}{\partial \lambda_2^2} = \exp(\lambda_2) V_\lambda^{-1} Q_2 V_\lambda^{-1} - 2\exp(2\lambda_2) V_\lambda^{-1} Q_2 V_\lambda^{-1} Q_2 V_\lambda^{-1}, \tag{S1.33}$$

and

$$\frac{\partial^2 V_\lambda^{-1}}{\partial x_1 \partial x_2} = \frac{\partial^2 A^{-1}}{\partial x_2 \partial x_1} = -\exp(\lambda_1+\lambda_2)\left(V_\lambda^{-2} Q_2 V_\lambda^{-1} + V_\lambda^{-1} Q_2 V_\lambda^{-2}\right). \tag{S1.34}$$

- $H_{\operatorname{tr}\left(V_\lambda^{-1} X S_\beta X^T\right)}$

Due to the linearity of the trace operator, we have

$$\frac{\partial^2 \operatorname{tr}\left(V_\lambda^{-1} X S_\beta X^T\right)}{\partial \lambda_i \partial \lambda_j} = \operatorname{tr}\left(\frac{\partial^2 V_\lambda^{-1}}{\partial \lambda_i \partial \lambda_j} X S_\beta X^T\right) \tag{S1.35}$$

for $i, j \in \{1, 2\}$. Thus we only have to use (S1.32) to (S1.34).

Notably, the evaluation of these Hessian matrices will necessitate the inversion of V_λ on every iteration of the optimization algorithm. This inversion can be performed efficiently using the diagonalized form of Q_2. As Q_2 is a real, symmetric matrix by design, there exists a diagonalized form given by $Q_2^D = P^T Q_2 P$, where P is a unitary transformation matrix ($P^T = P^{-1}$). The entries $l_i, i \in \{1, \ldots, n\}$ of Q_2^D are the eigenvalues of Q_2. We thus have

$$\begin{aligned} V_\lambda^{-1} &= \left(\exp(\lambda_1) I_n + \exp(\lambda_2) Q_2\right)^{-1} \\ &= \left(\exp(\lambda_1) P I_n P^T + \exp(\lambda_2) P Q_2^D P^T\right)^{-1} \\ &= \left(P\left(\exp(\lambda_1) I_n + \exp(\lambda_2) Q_2^D\right) P^T\right)^{-1} \\ &= P\left(\exp(\lambda_1) I_n + \exp(\lambda_2) Q_2^D\right)^{-1} P^T. \end{aligned} \tag{S1.36}$$

As $\exp(\lambda_1) I_n + \exp(\lambda_2) Q_2^D$ is a diagonal matrix, its inverse is easily evaluated, and the diagonalizing matrix P only needs to be computed once for any given Q_2.

The VB free energy update equations

In this section, we consider the iterative maximization of the VB free energy function with respect to its vector and matrix parameters $m_\beta, S_\beta, m_\lambda$ and S_λ. In each case, we identify the relevant subpart of the VB free energy function depending on the respective parameter, evaluate its gradient with respect to the parameter in question, set the gradient to zero, and, if possible, solve the ensuing equation for a parameter update equation. To emphasize the iterative character of this endeavour, we use the superscript (i) to denote the values of parameters at a given algorithm iteration.

We consider the update with respect to S_λ first. The relevant subpart of $F^{VB}\left(m_\beta^{(i)}, S_\beta^{(i)}, m_\lambda^{(i)}, S_\lambda^{(i)}\right)$ depending on S_λ is given by

$$f^{VB}(S_\lambda) = -\frac{1}{4}\operatorname{tr}\left(B_{m_\beta^{(i)}, S_\beta^{(i)}, m_\lambda^{(i)}} S_\lambda\right) - \frac{1}{2}\operatorname{tr}(\Sigma_\lambda^{-1} S_\lambda) + \frac{1}{2}\ln|S_\lambda|. \tag{S1.37}$$

Using the identities (S1.13), (S1.14), and considering that $B_{m_\beta^{(i)}, S_\beta^{(i)}, m_\lambda^{(i)}}$ and Σ_λ^{-1} are symmetric, evaluation of the gradient of f^{VB} results in

$$\nabla f^{VB}(S_\lambda) = -\frac{1}{4} B_{m_\beta^{(i)}, S_\beta^{(i)}, m_\lambda^{(i)}} - \frac{1}{2}\Sigma_\lambda^{-1} + \frac{1}{2} S_\lambda^{-1}. \tag{S1.38}$$

Setting the gradient to zero and solving for the parameter update $S_\lambda^{(i+1)}$ then yields

$$S_\lambda^{(i+1)} := \left(\frac{1}{2} B_{m_\beta^{(i)}, S_\beta^{(i)}, m_\lambda^{(i)}} + \Sigma_\lambda^{-1}\right)^{-1}. \tag{S1.39}$$

Note that with the linearity properties of the trace operator, this update equation implies as a result, that the sum of the two trace terms involving S_λ in the VB free energy (equation (28) of the main text) evaluates to $-\frac{k}{2}$

and the term $B_{m_\beta^{(i)},S_\beta^{(i)},m_\lambda^{(i)}}$ does not need to be considered when deriving the update equations for m_β, S_β, and m_λ.

Next, the relevant subpart of $F^{VB}\left(m_\beta^{(i)}, S_\beta^{(i)}, m_\lambda^{(i)}, S_\lambda^{(i+1)}\right)$ depending on m_β is given by

$$f^{VB}(m_\beta) = -\frac{1}{2}(y - Xm_\beta)^T V_{m_\lambda}^{-1}(y - Xm_\beta) - \frac{1}{2}(m_\beta - \mu_\beta)^T S_\beta^{-1}(m_\beta - \mu_\beta), \tag{S1.40}$$

where we omitted iteration superscripts for visual clarity. With (S1.2), the gradient of $f^{VB}(m_\beta)$ is given by

$$\begin{aligned}\nabla f^{VB}(m_\beta) &= (y - Xm_\beta)^T V_{m_\lambda}^{-1} X - (m_\beta - \mu_\beta)^T \Sigma_\beta^{-1} \\ &= y^T V_{m_\lambda}^{-1} X - m_\beta^T X^T V_{m_\lambda}^{-1} X - m_\beta^T \Sigma_\beta^{-1} + \mu_\beta^T \Sigma_\beta^{-1}\end{aligned} \tag{S1.41}$$

Setting the gradient to zero then yields the update equation

$$m_\beta^{(i+1)} := \left(X^T V_{m_\lambda}^{-1} X + \Sigma_\beta^{-1}\right)^{-1} \left(X^T V_{m_\lambda}^{-1} y + \Sigma_\beta^{-1} \mu_\beta\right) \tag{S1.42}$$

Analogously, the relevant subpart of $F^{VB}\left(m_\beta^{(i+1)}, S_\beta^{(i)}, m_\lambda^{(i)}, S_\lambda^{(i+1)}\right)$ depending on S_β is given by

$$f^{VB}(S_\beta) = -\frac{1}{2}\operatorname{tr}\left(X^T V_{m_\lambda}^{-1} X S_\beta\right) - \frac{1}{2}\operatorname{tr}(\Sigma_\beta^{-1} S_\beta) + \frac{1}{2}\ln|S_\beta| \tag{S1.43}$$

with gradient

$$\nabla f^{VB}(S_\beta) = -\frac{1}{2} X^T V_{m_\lambda}^{-1} X - \frac{1}{2}\Sigma_\beta^{-1} + \frac{1}{2} S_\beta^{-1} \tag{S1.44}$$

and the resulting update equation

$$S_\beta^{(i+1)} := \left(X^T V_{m_\lambda}^{-1} X + \Sigma_\beta^{-1}\right)^{-1}. \tag{S1.45}$$

Note that the update equations (S1.42) and (S1.45) conform to the well-known closed-form expressions for Bayesian inference in the conjugate Gaussian model (cf. eq. (9) of the main text), with the difference of the parametric dependence of the error covariance matrix on $m_\lambda^{(i)}$.

Finally, the relevant subpart of $F^{VB}\left(m_\beta^{(i+1)}, S_\beta^{(i+1)}, m_\lambda^{(i)}, S_\lambda^{(i+1)}\right)$ depending on m_λ is given by, again omitting iteration superscripts for visual clarity,

$$\begin{aligned}f^{VB}(m_\lambda) = &-\frac{1}{2}\ln|V_{m_\lambda}| - \frac{1}{2}(y - Xm_\beta)^T V_{m_\lambda}^{-1}(y - Xm_\beta) \\ &- \frac{1}{2}\operatorname{tr}(X^T V_{m_\lambda}^{-1} X S_\beta) - \frac{1}{2}(m_\lambda - \mu_\lambda)^T \Sigma_\lambda^{-1}(m_\lambda - \mu_\lambda).\end{aligned} \tag{S1.46}$$

Evaluation of entries $\frac{\partial}{\partial m_{\lambda_j}} f^{VB}(m_\lambda)$ of the gradient $\nabla f^{VB}(m_\lambda)$ yields

$$\begin{aligned}\frac{\partial}{\partial m_{\lambda_j}} f^{VB}(m_\lambda) = &-\frac{1}{2}\operatorname{tr}\left(V_{m_\lambda}^{-1}\left(\frac{\partial V_{m_\lambda}}{\partial m_{\lambda_j}}\right)\right) \\ &- \frac{1}{2}(y - Xm_\beta)^T \left(\frac{\partial V_{m_\lambda}^{-1}}{\partial m_{\lambda_j}}\right)(y - Xm_\beta) \\ &- \frac{1}{2}\operatorname{tr}\left(\left(\frac{\partial V_{m_\lambda}^{-1}}{\partial m_{\lambda_j}}\right) X S_\beta X^T\right) - \left((m_\lambda - \mu_\lambda)^T \Sigma_\lambda^{-1}\right)_j.\end{aligned} \tag{S1.47}$$

The evaluation of these entries for the two-component linear error covariance (S1.25) then yields

$$\begin{aligned}\frac{\partial}{\partial m_{\lambda_1}} f^{VB}(m_\lambda) = &-\frac{1}{2}\exp(m_{\lambda_1})\Big(\mathrm{tr}(V_{m_\lambda}^{-1}) - (y - Xm_\beta)^T V_{m_\lambda}^{-2}(y - Xm_\beta)\\ &- \mathrm{tr}\left(V_{m_\lambda}^{-1} X S_\beta X^T V_{m_\lambda}^{-1}\right)\Big) - \frac{1}{2}\left((m_\lambda - \mu_\lambda)\Sigma_\lambda^{-1}\right)_1,\end{aligned} \tag{S1.48}$$

and

$$\begin{aligned}\frac{\partial}{\partial m_{\lambda_2}} f^{VB}(m_\lambda) = &-\frac{1}{2}\exp(m_{\lambda_2})\big(\mathrm{tr}\left(V_{m_\lambda}^{-1}Q_2\right)\\ &- (y - Xm_\beta)^T V_{m_\lambda}^{-1} Q_2 V_{m_\lambda}^{-1}(y - Xm_\beta)\\ &- \mathrm{tr}\left(Q_2 V_{m_\lambda}^{-1} X S_\beta X^T V_{m_\lambda}^{-1}\right)\big) - \frac{1}{2}\left((m_\lambda - \mu_\lambda)\Sigma_\lambda^{-1}\right)_2.\end{aligned} \tag{S1.49}$$

Lastly, to determine the value $m_\lambda^{(i+1)}$ for which

$$\frac{\partial}{\partial m_{\lambda_j}} f^{VB}\left(m_\lambda^{(i+1)}\right) = 0 \tag{S1.50}$$

for $j = 1, 2$, we employ the routine *fsolve.m* provided by Matlab (MATLAB and Optimization Toolbox Release 2014b, The MathWorks, Inc., Natick, Massachusetts, United States). This function implements a trust-region dogleg algorithm for the minimization of nonlinear real-valued functions of multiple variables (Coleman and Li, 1996; Nocedal and Wright, 2006).

S1.3 Variational maximum likelihood

Evaluation of the VML free energy

The VML free energy is defined as

$$\begin{aligned}F^{VML}(q(\beta), \lambda) =& \langle \ln\left(\frac{p_\lambda(y, \beta)}{q(\beta)}\right)\rangle_{q(\beta)}\\ &= \langle \ln p_\lambda(y|\beta)\rangle_{q(\beta)} + \langle \ln p(\beta)\rangle_{q(\beta)} - \langle \ln q(\beta)\rangle_{q(\beta)}.\end{aligned} \tag{S1.51}$$

The latter two terms on the right-hand side of (S1.51) have been evaluated in Section S1.2. The first term can be evaluated using (S1.2), yielding

$$\begin{aligned}\langle \ln p_\lambda(y|\beta)\rangle_{q(\beta)} = &-\frac{n}{2}\ln 2\pi - \frac{1}{2}\ln|V_\lambda| - \frac{1}{2}(y - Xm_\beta)^T V_\lambda^{-1}(y - Xm_\beta)\\ &- \frac{1}{2}\mathrm{tr}(X^T V_\lambda^{-1} X S_\beta),\end{aligned} \tag{S1.52}$$

which completes the derivation of the VML free energy as eq. (39) of the main text.

The VML free energy update equations

To identify the update equations for the maximization of the VML free energy, we proceed as in Section S1.2. Because the main difference between the VB

and VML framework is the parameterization of the error covariance matrix V_λ in terms of λ rather than m_λ and the vanishing of terms relating to the prior and variational distributions of λ, we can keep the discussion very concise.

The relevant subpart of $F^{VML}(m_\beta^{(i)}, S_\beta^{(i)}, \lambda^{(i)})$ depending on m_β is given by

$$f^{VML}(m_\beta) = -\frac{1}{2}(y - Xm_\beta)^T V_\lambda^{-1}(y - Xm_\beta) - \frac{1}{2}(m_\beta - \mu_\beta)^T S_\beta^{-1}(m_\beta - \mu_\beta), \quad \text{(S1.53)}$$

with gradient

$$\nabla f^{VML}(m_\beta) = y^T V_\lambda^{-1} X - m_\beta^T X^T V_\lambda^{-1} X - m_\beta^T \Sigma_\beta^{-1} + \mu_\beta^T \Sigma_\beta^{-1} \quad \text{(S1.54)}$$

and ensuing update equation

$$m_\beta^{(i+1)} := \left(X^T V_\lambda^{-1} X + \Sigma_\beta^{-1}\right)^{-1} \left(X^T V_\lambda^{-1} X y + \Sigma_\beta^{-1} \mu_\beta\right). \quad \text{(S1.55)}$$

Likewise, the relevant subpart of $F^{VML}(m_\beta^{(i+1)}, S_\beta^{(i)}, \lambda^{(i)})$ depending on S_β is given by

$$f^{VML}(S_\beta) = -\frac{1}{2}\operatorname{tr}\left(V_\lambda^{-1} X S_\beta X^T\right) - \frac{1}{2}\operatorname{tr}(\Sigma_\beta^{-1} S_\beta) + \frac{1}{2}\ln|S_\beta| \quad \text{(S1.56)}$$

with gradient

$$\nabla f^{VML}(S_\beta) = -\frac{1}{2} X^T V_\lambda^{-1} X - \frac{1}{2}\Sigma_\beta^{-1} + \frac{1}{2} S_\beta^{-1} \quad \text{(S1.57)}$$

and the resulting update equation

$$S_\beta^{(i+1)} := \left(X^T V_\lambda^{-1} X + \Sigma_\beta^{-1}\right)^{-1}. \quad \text{(S1.58)}$$

Finally, the relevant subpart of $F^{VML}\left(m_\beta^{(i+1)}, S_\beta^{(i+1)}, \lambda^{(i)}\right)$ depending on λ is given by

$$f^{VML}(\lambda) = -\frac{1}{2}\ln|V_\lambda| - \frac{1}{2}(y - Xm_\beta)^T V_\lambda^{-1}(y - Xm_\beta) - \frac{1}{2}\operatorname{tr}\left(V_\lambda^{-1} X S_\beta X^T\right). \quad \text{(S1.59)}$$

Here, in analogy to eqs. (S1.48) and (S1.49), the entries of $\nabla f^{VML}(\lambda)$ for the case of the two-component error covariance matrix of interest (eq. (S1.25)) evaluate to

$$\begin{aligned} \frac{\partial}{\partial \lambda_1} f^{VML}(\lambda) &= -\frac{1}{2}\exp(\lambda_1)\left(\operatorname{tr}(V_\lambda^{-1}) - (y - Xm_\beta)^T V_\lambda^{-2}(y - Xm_\beta)\right) \\ &\quad + \frac{1}{2}\exp(\lambda_1)\operatorname{tr}\left(V_\lambda^{-2} X S_\beta X^T\right). \end{aligned} \quad \text{(S1.60)}$$

and

$$\begin{aligned} \frac{\partial}{\partial \lambda_2} f^{VML}(\lambda) &= -\frac{1}{2}\exp(\lambda_2)\left(\operatorname{tr}(V_\lambda^{-1} Q_2) - (y - Xm_\beta)^T V_\lambda^{-1} Q_2 V_\lambda^{-1}(y - Xm_\beta)\right) \\ &\quad + \frac{1}{2}\exp(\lambda_2)\operatorname{tr}\left(V_\lambda^{-1} Q_2 V_\lambda^{-1} X S_\beta X^T\right) \end{aligned} \quad \text{(S1.61)}$$

S1.4 Restricted maximum likelihood

The ReML objective function as VML free energy

We first show that for the probabilistic model

$$p_\lambda(y, \beta) = p_\lambda(y|\beta)p(\beta) \text{ with } p_\lambda(y|\beta) = N(y; X\beta, V_\lambda) \text{ and } p(\beta) := 1 \quad \text{(S1.62)}$$

it holds that the VML free energy with variational distribution

$$q(\beta) := p_\lambda(\beta|y) \quad \text{(S1.63)}$$

evaluates to the ReML objective function

$$\ell_{ReML}(\lambda) := -\frac{1}{2}\ln|V_\lambda| - \frac{1}{2}\ln|X^T V_\lambda^{-1} X| - \frac{1}{2}(y - X\hat{\beta}_{GLS})^T V_\lambda^{-1}(y - X\hat{\beta}_{GLS}) \quad \text{(S1.64)}$$

up to an additive constant, i.e.

$$F^{VML}(p_\lambda(\beta|y), \lambda) = \ell_{ReML}(\lambda) + c \quad \text{(S1.65)}$$

with

$$c := -\frac{n}{2}\ln(2\pi) + \frac{p}{2}\ln(2\pi) \quad \text{(S1.66)}$$

To this end, we first note that for the probabilistic model (S1.62) and with the definition of the GLS estimator

$$\hat{\beta}_{GLS} := \left(X^T V_\lambda^{-1} X\right)^{-1} X^T V_\lambda^{-1} y \quad \text{(S1.67)}$$

it holds that

$$p_\lambda(\beta|y) = N(\beta; m_\beta, S_\beta) = N\left(\beta; \hat{\beta}_{GLS}, \left(X^T V_\lambda^{-1} X\right)^{-1}\right). \quad \text{(S1.68)}$$

In brief, (S1.68) follows as a limiting case of the conditional properties of Gaussian distributions for the case of zero prior precision, i.e. the case of an improper prior $p(\beta) = 1$ (see e.g. Murphy (2012) for a more detailed discussion).

Evaluation of the VML free energy in the current scenario then yields

$$\begin{aligned} F^{VML}(p_\lambda(\beta|y), \lambda) &= \left\langle \ln\left(\frac{p_\lambda(y,\beta)}{p_\lambda(\beta|y)}\right)\right\rangle_{p_\lambda(\beta|y)} \\ &= \langle \ln\left(p_\lambda(y|\beta)p(\beta)\right)\rangle_{p_\lambda(\beta|y)} - \langle \ln p_\lambda(\beta|y)\rangle_{p_\lambda(\beta|y)} \\ &= \langle \ln p_\lambda(y|\beta)\rangle_{p_\lambda(\beta|y)} - \langle \ln p_\lambda(\beta|y)\rangle_{p_\lambda(\beta|y)}. \end{aligned} \quad \text{(S1.69)}$$

Evaluation of the first term on the right-hand side (S1.69) yields

$$
\begin{aligned}
\langle \ln p_\lambda(y|\beta)\rangle_{p_\lambda(\beta|y)} =& -\frac{n}{2}\ln 2\pi - \frac{1}{2}\ln|V_\lambda| - \frac{1}{2}\langle (y - X\beta)^T V_\lambda^{-1}(y - X\beta)\rangle_{p(\lambda)(\beta|y)} \\
=& -\frac{n}{2}\ln 2\pi - \frac{1}{2}\ln|V_\lambda| - \frac{1}{2}(y - X\hat{\beta}_{GLS})^T V_\lambda^{-1}(y - X\hat{\beta}_{GLS}) \\
& - \frac{1}{2}\operatorname{tr}\left(V_\lambda^{-1}X(X^T V_\lambda^{-1}X)^{-1}X^T\right) \\
=& -\frac{n}{2}\ln 2\pi - \frac{1}{2}\ln|V_\lambda| - \frac{1}{2}(y - X\hat{\beta}_{GLS})^T V_\lambda^{-1}(y - X\hat{\beta}_{GLS}) \\
& - \frac{1}{2}\operatorname{tr}\left(X^T V_\lambda^{-1}X(X^T V_\lambda^{-1}X)^{-1}\right) \\
=& -\frac{n}{2}\ln 2\pi - \frac{1}{2}\ln|V_\lambda| - \frac{1}{2}(y - X\hat{\beta}_{GLS})^T V_\lambda^{-1}(y - X\hat{\beta}_{GLS}) \\
& - \frac{p}{2},
\end{aligned}
\tag{S1.70}
$$

where the second equality follows with (S1.2). The third equality uses the invariance of the trace under cyclic permutations. The second term on the right hand of (S1.69) corresponds to the entropy of the distribution $p_\lambda(\beta|y)$ and thus evaluates to

$$
H(p_\lambda(\beta|y)) = -\langle p_\lambda(\beta|y)\rangle_{p_\lambda(\beta|y)} = \frac{p}{2}\ln(2\pi e) + \ln|S_\beta| = \frac{p}{2}\ln(2\pi e) - \frac{1}{2}\ln|X^T V_\lambda^{-1}X|
\tag{S1.71}
$$

We thus have shown that

$$
F^{VML}(p_\lambda(\beta|y),\lambda) = \ell_{ReML}(\lambda) - \frac{n}{2}\ln 2\pi + \frac{p}{2}\ln(2\pi e) - \frac{p}{2},
\tag{S1.72}
$$

which concludes the derivation.

Evaluation of the ReML free energy function

To align the discussion of ReML with the previous discussions of VB and VML, we next define the ReML free energy function as the VML free energy evaluated for the probabilistic model (S1.62) at the exact posterior distribution $p_\lambda(\beta|y)$, i.e.,

$$
F^{ReML}(m_\beta, S_\beta, \lambda) := F^{VML}(p_\lambda(\beta|y),\lambda) = \ell_{ReML}(\lambda) + c.
\tag{S1.73}
$$

By noting that with (S1.68) the variational parameters are given by

$$
m_\beta = \hat{\beta}_{GLS} \text{ and } S_\beta = (X^T V_\lambda^{-1}X)^{-1},
\tag{S1.74}
$$

we can then rewrite the ReML free energy as in the main text:

$$
\begin{aligned}
F^{ReML}(m_\beta, S_\beta, \lambda) &= -\frac{1}{2}\ln|V_\lambda| - \frac{1}{2}\ln|X^T V_\lambda^{-1} X| \\
&\quad - \frac{1}{2}(y - X\hat{\beta}_{GLS})^T V_\lambda^{-1}(y - X\hat{\beta}_{GLS}) \\
&\quad - \frac{n}{2}\ln 2\pi + \frac{p}{2}\ln(2\pi e) - \frac{p}{2} \\
&= -\frac{1}{2}\ln|V_\lambda| + \frac{1}{2}\ln|(X^T V_\lambda^{-1} X)^{-1}| \\
&\quad - \frac{1}{2}(y - Xm_\beta)^T V_\lambda^{-1}(y - Xm_\beta) \\
&\quad - \frac{n}{2}\ln 2\pi + \frac{p}{2}\ln(2\pi e) - \frac{1}{2}\operatorname{tr}\left((X^T V_\lambda^{-1} X)(X^T V_\lambda^{-1} X)^{-1}\right) \\
&= -\frac{1}{2}\ln|V_\lambda| + \frac{1}{2}\ln|S_\beta| \\
&\quad - \frac{1}{2}(y - Xm_\beta)^T V_\lambda^{-1}(y - Xm_\beta) \\
&\quad - \frac{n}{2}\ln 2\pi + \frac{p}{2}\ln(2\pi e) - \frac{1}{2}\operatorname{tr}(S_\beta X^T V_\lambda^{-1} X) \\
&= -\frac{n}{2}\ln 2\pi - \frac{1}{2}\ln|V_\lambda| - \frac{1}{2}(y - Xm_\beta)^T V_\lambda^{-1}(y - Xm_\beta) \\
&\quad - \frac{1}{2}\operatorname{tr}(S_\beta X^T V_\lambda^{-1} X) \\
&\quad + \frac{p}{2}\ln(2\pi e) + \frac{1}{2}\ln|S_\beta|.
\end{aligned}
\tag{S1.75}
$$

The ReML free energy update equations

Finally, we derive the update equations for the parameters m_β, S_β, and λ of the ReML free energy. Note that because the ReML objective function is identical to the ReML free energy up to an additive constant which is independent of these parameters, the resulting iterative algorithm also maximizes the ReML objective function.

The relevant subpart of $F^{ReML}(m_\beta^{(i)}, S_\beta^{(i)}, \lambda^{(i)})$ that depends on m_β is given by, omitting iteration superscripts for ease of notation,

$$f^{ReML}(m_\beta) = -\frac{1}{2}(y - Xm_\beta)^T V_\lambda^{-1}(y - Xm_\beta). \tag{S1.76}$$

with gradient

$$\nabla f^{ReML}(m_\beta) = y^T V_\lambda^{-1} X - m_\beta^T X^T V_\lambda^{-1} X \tag{S1.77}$$

and ensuing update equation

$$m_\beta^{(i+1)} := (X^T V_\lambda^{-1} X)^{-1} X^T V_\lambda^{-1} y. \tag{S1.78}$$

Unsurprisingly, this is the GLS estimator. Further, the relevant subpart of $F^{ReML}(m_\beta^{(i+1)}, S_\beta^{(i)}, \lambda^{(i)})$ depending on S_β is given by, again omitting iteration superscripts for ease of notation,

$$f^{ReML}(S_\beta) = -\frac{1}{2}\operatorname{tr}(S_\beta X^T V_\lambda^{-1} X) + \frac{1}{2}\ln|S_\beta| \tag{S1.79}$$

with gradient

$$\nabla f^{ReML}(S_\beta) = -\frac{1}{2}X^T V_\lambda^{-1} X + \frac{1}{2}S_\beta^{-1} \quad \text{(S1.80)}$$

and ensuing update equation

$$S_\beta^{(i+1)} := (X^T V_\lambda^{-1} X)^{-1}. \quad \text{(S1.81)}$$

Finally, because the subpart of F^{ReML} depending on λ is identical to the subpart of F^{VML} depending on λ, the update procedure for F^{ReML} with respect to λ is identical to that of F^{VML}.

S1.5 Maximum likelihood

The ML free energy update equations

For the GLM, we have by definition

$$F^{ML}(\beta, \lambda) = -\frac{n}{2}\ln(2\pi) - \frac{1}{2}\ln|V_\lambda| - \frac{1}{2}(y - X\beta)^T V_\lambda^{-1}(y - X\beta) \quad \text{(S1.82)}$$

To derive parameter update equations, we consider the dependency of F^{ML} on $\beta^{(i)}$ and $\lambda^{(i)}$ in turn. The relevant subpart of $F^{ML}(\beta^{(i)}, \lambda^{(i)})$ that depends on β is then given by, omitting iteration superscripts for ease of notation,

$$f^{ML}(\beta) = -\frac{1}{2}(y - X\beta)^T V_\lambda^{-1}(y - X\beta) \quad \text{(S1.83)}$$

with gradient

$$\nabla f^{ML}(\beta) = y^T V_\lambda^{-1} X - \beta^T X^T V_\lambda^{-1} X \quad \text{(S1.84)}$$

and ensuing update equation

$$\beta^{(i+1)} := (X^T V_\lambda^{-1} X)^{-1} X^T V_\lambda^{-1} y, \quad \text{(S1.85)}$$

corresponding to the GLS estimator as in the case of ReML. The relevant subpart of $F^{ML}(\beta^{(i+1)}, \lambda^{(i)})$ that depends on λ differs from the VML and ReML scenarios and is given by, again omitting iteration superscripts for ease of notation,

$$f^{ML}(\lambda) = -\frac{1}{2}\ln|V_\lambda| - \frac{1}{2}(y - X\beta)^T V_\lambda^{-1}(y - X\beta) \quad \text{(S1.86)}$$

Here, in analogy to eqs. (S1.47), (S1.48), and (S1.49), the entries of $\nabla f^{ML}(\lambda)$ for the case of the two-component error covariance matrix of interest evaluate to

$$\frac{\partial}{\partial \lambda_1} f^{ML}(\lambda) = -\frac{1}{2}\exp(\lambda_1)\left(\mathrm{tr}(V_\lambda^{-1}) - (y - X\beta)^T V_\lambda^{-2}(y - X\beta)\right) \quad \text{(S1.87)}$$

and

$$\frac{\partial}{\partial \lambda_2} f^{VB}(\lambda) = -\frac{1}{2}\exp(\lambda_2)\left(\mathrm{tr}(V_\lambda^{-1}Q_2) - (y - X\beta)^T V_\lambda^{-1} Q_2 V_\lambda^{-1}(y - X\beta)\right) \quad \text{(S1.88)}$$

As they correspond to a disregard of prior information and posterior uncertainty about β, equations (S1.85), (S1.87) and (S1.88) can also be attained from the VML update equations (S1.55), (S1.60) and (S1.61) by setting $\Sigma_\beta^{-1} = S_\beta = 0$.

S2 Foundations of variational Bayes

In this section we formulate a probability-theoretic model of the probabilistic model considered in the main text in order to derive the VML and ML scenarios as special cases of VB. By "probability-theoretic" we mean a measure theory-based approach to probabilistic concepts, as prevalent in contemporary mathematics (e.g. Billingsley, 2012; Shao, 2003; Fristedt and Gray, 1997). This approach is rather uncommon in the neuroimaging and machine learning literature, where many application-oriented developments on VB have taken place (Blei et al., 2016). In the current context, it is necessitated by the fact that "point probability masses" cannot be represented by probability density functions. This implies that to derive VML and ML under VB requires a careful differentiation between those random variables whose distribution can and cannot be represented by probability density functions. This is afforded by the measure theory-based approach. We assume that the reader is familiar with the measure-theoretic viewpoint of probability theory, including Lebesgue integration. To establish notation and prepare some aspects of the discussion to follow, we provide a brief summary of key elements in Section S2.1. In Section S2.2 we then review a selection of entropy formulations which will be required for the formulation of VB and VML in probability-theoretic terms. Finally, in Section S2.3 we formulate the VB, VML, and ML scenarios is probability-theoretic terms and discuss their mutual relationships.

S2.1 Preliminaries

Measurable, measure, and probability spaces

Our formulation rests on the concepts of *measurable*, *measure*, and *probability spaces*. A *measurable space* is a pair $(\Omega, \mathcal{F})$, where Ω denotes a set and $\mathcal{F}$ denotes a σ-field on Ω. An important measurable space in the following will be $(\mathbb{R}^d, \mathcal{B}^d)$, where $\mathcal{B}^d$ denotes the d-dimensional Borel σ-field on $\mathbb{R}^d$. A *measure space* is a triple $(\Omega, \mathcal{F}, \mu)$, where μ denotes a measure, i.e. a mapping $\mu : \mathcal{F} \to [0, \infty]$ with properties

(M1) $\mu(\emptyset) = 0$, and

(M2) for every pairwise disjoint sequence $\{A_i\}_{i \in \mathbb{N}}$ with $A_i \in \mathcal{F}, i \in \mathbb{N}$ it holds that $\mu\left(\cup_{i=1}^{\infty} A_i\right) = \sum_{i=1}^{\infty} \mu(A_i)$.

An important measure space in the following will be $(\mathbb{R}^d, \mathcal{B}^d, \mu_l^d)$, where $\mathcal{B}^d$ denotes the d-dimensional *Borel σ-field* and μ_l^d denotes the d-dimensional *Lebesgue measure*

$$\mu_l^d : \mathcal{B}^d \to [0, \infty], \times_{i=1}^d [a_i, b_i[\mapsto \mu_l^d \left(\times_{i=1}^d [a_i, b_i[\right) := \prod_{i=1}^d (b_i - a_i). \tag{S2.1}$$

Please note that we do not use the more conventional notation λ^d for the Lebesgue measure to avoid confusion with the covariance component parameter vector λ. Similarly, a *probability space* is a triple $(\Omega, \mathcal{F}, P)$, where P denotes a probability measure, i.e. a mapping $P : \mathcal{F} \to [0, 1]$, with properties

(P1) $P(\emptyset) = 0, P(\Omega) = 1$, and

(P2) for every pairwise disjoint sequence $\{A_i\}_{i\in\mathbb{N}}$ with $A_i \in \mathcal{F}, i \in \mathbb{N}$ it holds that $P(\cup_{i=1}^{\infty} A_i) = \sum_{i=1}^{\infty} P(A_i)$.

An important probability space in the following will be $(\mathbb{R}^d, \mathcal{B}^d, \delta_x)$, where δ_x denotes the *Dirac measure*, defined as

$$\delta_x : \mathcal{B}^d \to [0,1], B \mapsto \delta_x(B) := \begin{cases} 1 \text{ if } x \in B \\ 0 \text{ if } x \notin B \end{cases} . \tag{S2.2}$$

Of key importance in the derivations to follow is the fact that the Lebesgue integral of a measurable function $f : \mathbb{R}^d \to \mathbb{R}^d$ with respect to the Dirac measure δ_x is readily evaluated as (e.g. Lieb and Loss, 2001)

$$\int f \, d\delta_x = f(x). \tag{S2.3}$$

Random variables and distributions

Let $(\Omega, \mathcal{F}, P)$ and $(\Gamma, \mathcal{S})$ denote a probability space and a measurable space, respectively. A *random variable* is a function

$$X : (\Omega, \mathcal{F}, P) \to (\Gamma, \mathcal{S}), \tag{S2.4}$$

which is measurable, i.e. for which

$$X^{-1}(S) := \{\omega \in \Omega | X(\omega) \in S\} \in \mathcal{F} \text{ for all } S \in \mathcal{S}. \tag{S2.5}$$

A random variable induces a probability measure

$$P_X : \mathcal{S} \to [0,1], S \mapsto P_X(S) := P(\{\omega \in \Omega | X(\omega) \in S\}), \tag{S2.6}$$

on $\mathcal{S}$. The probability measure P_X is referred to as the *distribution* of the random variable X and renders $(\Gamma, \mathcal{S}, P_X)$ a probability space (e.g. Fristedt and Gray, 1997, Chapter 2).

Probability density functions

For a measure space $(\Omega, \mathcal{F}, \mu)$ any quasiintegrable function $f : \Omega \to \mathbb{R}_{\geq 0}$ is a *density function* and defines a measure ν on $\mathcal{F}$ by means of its Lebesgue integral for $A \in \mathcal{F}$, i.e.

$$\nu : \mathcal{F} \to [0, \infty], A \mapsto \nu(A) := \int_A f \, d\mu. \tag{S2.7}$$

We say that "ν is a measure with density function f with respect to the measure μ" and write $\nu = f\mu$ for short. Recall that a $\mathcal{F}$-measurable function $g : \Omega \to \bar{\mathbb{R}}$ is integrable with respect to ν, if the function product $g \cdot f$ is integrable with respect to μ, and that in this case

$$\int_\Omega g \, d\nu = \int_\Omega g \cdot f \, d\mu \tag{S2.8}$$

(e.g. Billingsley, 2012, Theorem 16.11). As noted above, we will be primarily concerned with the measure space $(\mathbb{R}^d, \mathcal{B}^d, \mu_l^d)$, where μ_l^d denotes the Lebesgue measure on $\mathbb{R}^d$. In this case, if the Lebesgue integral of f with respect to μ_l^d equals 1, f is referred to as *probability density function*. Furthermore, in this case we have for $\nu = f\mu_l^d$

$$\int g\, d\nu = \int g \cdot f\, d\mu_l^d = \int g(x)f(x)\, dx. \tag{S2.9}$$

Crucially, this implies that one can evaluate Lebesgue integrals using Riemann integration as done throughout Section S1 (right-hand side of (S2.9), for details see e.g. Schmidt (2011), Chapter 9).

We further require the notion of conditional probability density functions. For a random variable (X, Y) on a product measure space $(\Omega, \mathcal{F}, \mu) := (\Omega_x \times \Omega_y, \mathcal{F}_x \times \mathcal{F}_y, \mu_x \times \mu_y)$ with joint probability density function $f_{X,Y} : \Omega \to \mathbb{R}_{>0}$ with respect to μ, the conditional probability density function of X given $Y = y$ is defined as

$$f_{X|Y} : \Omega \to \mathbb{R}_{>0}, (x, y) \mapsto f_{X|Y}(x, y) := f_{X,Y}(x, y)/f_Y(y) \tag{S2.10}$$

where

$$f_Y : \Omega_2 \to \mathbb{R}_{>0}, y \mapsto f_Y(y) := \int f_{X,Y}(x, y)\, d\mu_x \tag{S2.11}$$

is the marginal probability density function of Y with respect to μ_y (e.g. Shao, 2003, Chapter 1.4).

Discrete, continuous, and mixed random vectors

Because we are considering multivariate random entities in the application of VB, VML, and ML to the GLM, we also require the notion of *random vectors* as the multivariate extension of random variables. More specifically, we require the concepts of *discrete, continuous* and *mixed random vectors*, which we introduce in the following.

Let $(\Omega, \mathcal{F}, P)$ be a probability space. A d-dimensional *discrete random vector* is a function

$$X : \Omega \to \mathbb{R}^d, \omega \mapsto X(\omega) := (X_1(\omega), ..., X_d(\omega))^T \tag{S2.12}$$

whose range space or *alphabet* (Gray, 2011; Cover and Thomas, 2012)

$$\mathcal{X} := \{x_i\}_{i=1}^n := X(\Omega) \subset \mathbb{R}^d \tag{S2.13}$$

is finite. A discrete random vector has an associated *probability mass function* p_X given by

$$p_X : \mathbb{R}^d \to [0, 1], p_X(x) := \begin{cases} P(X = x), & \text{if } x \in \mathcal{X} \\ 0, & \text{if } x \notin \mathcal{X} \end{cases}, \tag{S2.14}$$

with the notational convention

$$P(X = x) := P(\{X = x\}) = P(\{\omega \in \Omega | X(\omega) = x\}). \tag{S2.15}$$

A special discrete random vector required in the following is a *constant random vector*, which for a fixed $x^* \in \mathbb{R}^d$ we write as

$$X^* : \Omega \to \mathbb{R}^d, \omega \mapsto X^*(\omega) := x^*. \tag{S2.16}$$

In this case the alphabet $\mathcal{X}^* = \{x^*\}$ of X^* comprises a single element, the associated probability mass of which is given by

$$p_{X^*}(x^*) = P(\{\omega \in \Omega | X^*(\omega) = x^*)\}) = P(\Omega) = 1. \tag{S2.17}$$

Analogously to a random variable, a random vector $X : \Omega \to \mathbb{R}^d$ induces a probability measure P_X on the measurable space $(\mathbb{R}^d, \mathcal{B}^d)$. Notably, the induced probability measures of a constant random vector is the Dirac measure (e.g. Bauer, 1991, p. 25), i.e. with (S2.2) and (S2.16)

$$P_{X^*} = \delta_{x^*}. \tag{S2.18}$$

A d-dimensional *continuous random vector* is a function Y of the form (S2.12) whose induced probability measure P_Y on $(\mathbb{R}^d, \mathcal{B}^d)$ is absolutely continuous with respect to Lebesgue measure and can thus be represented by a probability density function $f_Y : \mathbb{R}^d \to \mathbb{R}_{\geq 0}$.

Finally, we construct the concept of a *mixed random vector* as follows. Set $d := d_1 + d_2$ $(d_1, d_2 \in \mathbb{N})$, let $(\Omega, \mathcal{F})$ be a measurable space, and let $X : \Omega \to \mathbb{R}^{d_1}$ be a discrete random vector, which induces a distribution P_X on $\mathcal{P}(\mathcal{X})$. Let $P_{Y|X}$ be a Markov kernel from $(\mathcal{X}, \mathcal{P}(\mathcal{X}))$ to $(\mathbb{R}^{d_2}, \mathcal{B}^{d_2})$, i.e. $P_{Y|X}$ is a mapping

$$P_{Y|X} : \mathcal{X} \times \mathcal{B}^{d_2} \to \mathbb{R} \tag{S2.19}$$

with the properties

- $P_{Y|X}(x, \cdot) : \mathcal{B}^{d_2} \to [0, 1]$ is a probability measure on $\mathcal{B}^{d_2}$ for every $x \in \mathcal{X}$,
- $P_{Y|X}(\cdot, B) : \mathcal{X} \to \mathbb{R}$ is $\mathcal{P}(\mathcal{X})$-measurable for every $B \in \mathcal{B}^{d_2}$.

Then $P_{X,Y} = P_X P_{Y|X}$ is a probability measure on $(\Omega \times \mathbb{R}^{d_2}, \mathcal{F} \otimes \mathcal{B}^{d_2})$ (e.g. Shao, 2003, 1.4.3). Assume in addition that

$$P_{Y|X=x} := P_{Y|X}(x, \cdot) = f_{Y|X=x} \mu_l^{d_2}, \tag{S2.20}$$

i.e.

$$P_{Y|X=x}(B) = \int_B f_{Y|X=x}(y)\, dy \tag{S2.21}$$

for probability density functions $f_{Y|X=x}$ with $x \in \mathcal{X}$. Let Y denote the identity mapping on $\mathbb{R}^{d_2}$ and define $Z := (X, Y)$. Then Z is a d-dimensional random vector on $(\Omega \times \mathbb{R}^{d_2}, \mathcal{F} \otimes \mathcal{B}^{d_2})$ with marginal distributions having the properties

$$P_X(A) = P_{X,Y}(A \times \mathbb{R}^{d_2}) = \int_A P_{Y|X}(x, \mathbb{R}^{d_2})\, dP_X = \sum_{x \in A} p_X(x) \tag{S2.22}$$

for all $A \in \mathcal{P}(\mathcal{X})$ and

$$P_Y(B) = \sum_{x \in \mathcal{X}} p_X(x) \int_B f_{Y|X=x}(y)\, dy = \int_B \sum_{x \in \mathcal{X}} p_X(x) f_{Y|X=x}(y)\, dy =: \int_B f_Y(y)\, dy \tag{S2.23}$$

for all $B \in \mathcal{B}^{d_2}$. In other words, X is (by definition) a discrete d_1-dimensional random vector (which we call the *discrete component* of Z) and Y is (by construction) a continuous d_2-dimensional random vector (which we call the *continuous component* of Z). We call Z a d-dimensional *mixed random vector*.

Note that if we set $P_{Y|X}(x, \cdot) := P_Y$ for every $x \in \mathcal{X}$ with a probability measure P_Y on $\mathcal{B}^{d_2}$, then $P_{X,Y} = P_X P_Y$ is a product measure and the random vectors X and Y are independent. Vice versa, assuming independent d_1- and d_2-dimensional random vectors X and Y, respectively, we can use the construction above to construct a d-dimensional mixed random vector with discrete and continuous components whose marginal distributions are independent.

S2.2 Entropies of distributions of random vectors

Entropy of the distributions of a discrete random vector

Following (Gray, 2011, Chapter 3), we define the entropy of the distribution P_X of a discrete random vector X with alphabet $\mathcal{X}$ as

$$H(P_X) := -\sum_{x \in \mathcal{X}} p_X(x) \ln p_X(x) \tag{S2.24}$$

with the convention $0 \ln 0 := 0$. For later reference we note that the the entropy of the distribution of a constant random vector of the form (S2.16) is zero, because in this case the defining sum (S2.24) comprises a single term which evaluates to zero:

$$H(P_{X^*}) = -p_{X^*}(x^*) \ln p_{X^*}(x^*) = -1 \ln 1 = 0. \tag{S2.25}$$

Entropy of the distribution of a continuous random vector

We define the entropy of the distribution P_Y of a continuous random vector Y as its *differential entropy* (e.g. Cover and Thomas, 2012, Chapter 8), i.e. we set

$$h(P_Y) := -\int_{\mathbb{R}^d} f_Y(y) \ln f_Y(y)\, dy. \tag{S2.26}$$

Entropy of the distribution of a mixed random vector

Finally, following (Nair et al., 2006), we define the entropy of the distribution P_Z of a mixed random vector $Z = (X, Y)$ with the property

$$\int_{\mathbb{R}^{d_2}} |f_{Y|X=x}(y) \ln f_{Y|X=x}(y)| dy < \infty \tag{S2.27}$$

for all $x \in \mathcal{X}$ by

$$\mathcal{H}(P_Z) := -\sum_{x \in \mathcal{X}} \int_{\mathbb{R}^{d_2}} p_X(x) f_{Y|X=x}(y) \ln \left(p_X(x) f_{Y|X=x}(y) \right) dy \tag{S2.28}$$

Note that we can rewrite this definition as

$$\mathcal{H}(P_Z) = -\sum_{x\in\mathcal{X}} p_X(x)\ln p_X(x) - \sum_{x\in\mathcal{X}} p_X(x)\int_{\mathbb{R}^{d_2}} f_{Y|X=x}(y)\ln(f_{Y|X=x}(y))\,dy. \tag{S2.29}$$

$\mathcal{H}(P_Z)$ thus comprises the sum of the entropy of the marginal distribution of the discrete component of Z and a convex combination of the differential entropies of the conditional distributions of the continuous components.

In particular, for the case of independent discrete and continuous components, i.e. $f_{Y|X=x} := f_Y$, we obtain

$$\mathcal{H}(P_Z) = H(P_X) + h(P_Y). \tag{S2.30}$$

More generally, if the components X and Y are independent, and Y may be either continuous or discrete (rendering Z a discrete random vector), we write

$$\mathcal{H}(P_Z) = H(P_X) + \mathbb{H}(P_Y), \tag{S2.31}$$

where $\mathbb{H}$ denotes the entropy of the continuous or discrete component Y.

S2.3 Probability-theoretic variational Bayes

Based on the concepts reviewed in Sections S2.1 and S2.2, we are now in the position to formulate the VB, VML, and ML scenarios discussed in the main text in probability-theoretic terms and to delineate their relationship. We proceed as follows. First, we reformulate the free energy functions F^{VB}, F^{VML} and F^{ML} introduced in the main text in probability-theoretic terms. To distinguish these functions from their counterparts in the main text, they will be denoted by $\mathbb{F}^{VB}, \mathbb{F}^{VML}$, and $\mathbb{F}^{ML}$, respectively. Note that in general, the free energy functions depend on both the realizations of the observed data random variables as well as the distributions (or values in the VML and ML case) of the unobserved parameter random variables (or non-random variables in the VML and ML case). However, in analogy to classical likelihood functions (e.g. Shao, 2003, Chapter 4.4), we conceive of the free energy functions as functions of entities related to the parameter (random) variables only. Intuitively, this corresponds to assumption of a given and fixed data observation - the common scenario in experimental applications of the approaches. Second, upon reformulating the VB, VML, and ML scenarios in probability-theoretic terms, we relate these new formulations to the definitions of the variational free energies in the main text and show their consistency. Finally, we conclude with a theorem on the relationshiop between VB, VML, and ML.

Variational Bayes

To express the VB scenario of the main text in probability-theoretic terms, we set $d_1 := n+p+k$ $(n, p, k \in \mathbb{N})$, consider a probability space $(\Omega, \mathcal{F}, P)$ and the measurable space $(\mathbb{R}^{d_1}, \mathbb{B}^{d_1})$ and define the continuous random vector

$$(Y, B, L) : \Omega \to \mathbb{R}^{d_1}, \omega \mapsto (Y, B, L)(\omega), \tag{S2.32}$$

which induces a probability measure $P_{Y,B,L}$ on $\mathcal{B}^{d_1}$. We thus obtain the probability space $(\mathbb{R}^{d_1}, \mathcal{B}^{d_1}, P_{Y,B,L})$. Because (Y, B, L) is a continuous random vector, $P_{Y,B,L}$ can be represented by a probability density function

$$f_{Y,B,L} : \mathbb{R}^{d_1} \to \mathbb{R}_{>0}, (y, \beta, \lambda) \mapsto f_{Y,B,L}(y, \beta, \lambda) \tag{S2.33}$$

with respect to Lebesgue measure $\mu_l^{d_1}$ on $\mathcal{B}^{d_1}$. Note that $f_{Y,B,L}$ is denoted by $p(y, \beta, \lambda)$ in the main text.

To define the variational Bayes free energy function $\mathbb{F}^{VB}$, we first consider a random vector

$$(\tilde{B}, \tilde{L}) : \Omega \to \mathbb{R}^{p+k},\ \omega \mapsto (\tilde{B}, \tilde{L})(\omega) \tag{S2.34}$$

whose components are the independent random vectors $\tilde{B} : \Omega \to \mathbb{R}^p$ and $\tilde{L} : \Omega \to \mathbb{R}^k$. This implies that the induced distribution $Q_{(\tilde{B},\tilde{L})}$ on the measurable space $(\mathbb{R}^{p+k}, \mathcal{B}^{p+k})$ of $(\tilde{B}, \tilde{L})$ factorizes, i.e.

$$Q_{(\tilde{B},\tilde{L})} = Q_{\tilde{B}} \otimes Q_{\tilde{L}} \tag{S2.35}$$

where $Q_{\tilde{B}}$ and $Q_{\tilde{L}}$ denote the marginal distribution on $\mathcal{B}^p$ and $\mathcal{B}^k$ induced by $\tilde{B}$ and $\tilde{L}$, respectively (e.g. Fristedt and Gray, 1997, Chapter 9). We hence write $Q_{\tilde{B}\otimes\tilde{L}}$ for $Q_{(\tilde{B},\tilde{L})}$. Let $\mathcal{Q}_{\tilde{B}\otimes\tilde{L}}$ denote the set of all such distributions. For a fixed $y \in \mathbb{R}^n$ we then define

$$\mathbb{F}^{VB} : \mathcal{Q}_{\tilde{B}\otimes\tilde{L}} \to \mathbb{R}, Q_{(\tilde{B}\otimes\tilde{L})} \mapsto \mathbb{F}^{VB}(Q_{\tilde{B}\otimes\tilde{L}}) := \int \ln f_{Y,B,L}(y, \cdot, \cdot)\, dQ_{\tilde{B}\otimes\tilde{L}} + \mathbb{H}(Q_{\tilde{B}\otimes\tilde{L}}). \tag{S2.36}$$

Here the symbol $\mathbb{H}$ denotes the entropy of the distribution $Q_{\tilde{B}\otimes\tilde{L}}$, the evaluation of which depends on the type of random vectors $\tilde{B}$ and $\tilde{L}$, as discussed in Section S2.2.

Variational maximum likelihood

In analogy to the above, we set $d_2 := n + p$ $(n, p \in \mathbb{N})$, consider a probability space $(\Omega, \mathcal{F}, P)$ and the measurable space $(\mathbb{R}^{d_2}, \mathcal{B}^{d_2})$, and define the continuous random vector

$$(Y, B) : \Omega \to \mathbb{R}^{d_2}, \omega \mapsto (Y, B)(\omega), \tag{S2.37}$$

which induces the probability measure $P_{Y,B}$ on $\mathcal{B}^{d_2}$. We thus obtain the probability space $(\mathbb{R}^{d_2}, \mathcal{B}^{d_2}, P_{Y,B})$. As in the main text, we assume that $P_{Y,B}$ is represented by a parameter-dependent probability density function

$$f_{Y,B}^{\lambda} : \mathbb{R}^{d_2} \to \mathbb{R}_{>0}, (y, \beta) \mapsto f_{Y,B}^{\lambda}(y, \beta) \tag{S2.38}$$

with respect to Lebesgue measure on $\mathcal{B}^{d_2}$. Note that $f_{Y,B}^{\lambda}$ is denoted by $p_\lambda(y, \beta)$ in the main text.

To define the variational maximum likelihood free energy function $\mathbb{F}^{VML}$, we first consider a random vector $\tilde{B}$

$$\tilde{B} : \Omega \to \mathbb{R}^p, \omega \mapsto \tilde{B}(\omega), \tag{S2.39}$$

which induces a probability measure $Q_{\tilde{B}}$ on the measurable space $(\mathbb{R}^p, \mathcal{B}^p)$. Let $\mathcal{Q}_{\tilde{B}}$ denote the set of all such induced probability measures. For a fixed $y \in \mathbb{R}^n$ we then define

$$\mathbb{F}^{VML} : \mathcal{Q}_{\tilde{B}} \times \mathbb{R}^k \to \mathbb{R},\, (Q_{\tilde{B}}, \lambda) \mapsto \mathbb{F}^{VML}(Q_{\tilde{B}}, \lambda) := \int \ln f^{\lambda}_{Y,B}(y, \cdot) dQ_{\tilde{B}} + \mathbb{H}(Q_{\tilde{B}}), \quad \text{(S2.40)}$$

where as above the symbol $\mathbb{H}$ denotes the entropy of the distribution $Q_{\tilde{B}}$, the evaluation of which depends on the type of random vector $\tilde{B}$ as discussed in Section S2.2.

Maximum likelihood

Finally, to express the maximum likelihood scenario in probability-theoretic terms, we consider a probability space $(\Omega, \mathcal{F}, P)$, the measurable space $(\mathbb{R}^n, \mathcal{B}^n)$ and define the continuous random vector

$$Y : \Omega \to \mathbb{R}^n, \omega \mapsto Y(\omega), \quad \text{(S2.41)}$$

which induces the probability measure P_Y on $\mathcal{B}^n$ and hence the probability space $(\mathbb{R}^n, \mathcal{B}^n, P_Y)$. As in the main text, we assume that P_Y is represented by a parameter-dependent probability density function

$$f^{\beta,\lambda}_Y : \mathbb{R}^n \to \mathbb{R}_{>0}, y \mapsto f^{\beta,\lambda}_Y(y) \quad \text{(S2.42)}$$

with respect to Lebesgue measure on $\mathcal{B}^n$. Note that $f^{\beta,\lambda}_Y$ is denoted by $p_{\beta,\lambda}(y)$ in the main text. We define the maximum likelihood free energy function $\mathbb{F}^{ML}$ as the standard log-likelihood function of the maximum likelihood scenario, i.e. for fixed $y \in \mathbb{R}^n$, we set

$$\mathbb{F}^{ML} : \mathbb{R}^p \times \mathbb{R}^k \to \mathbb{R}, (\beta, \lambda) \mapsto \mathbb{F}^{ML}(\beta, \lambda) := \ln f^{\beta,\lambda}_Y(y). \quad \text{(S2.43)}$$

Note that we have $\mathbb{F}^{ML} = F^{ML}$ by definition.

This concludes the probability-theoretic formulations of the VB, VML, and ML, scenarios. We next show the consistency of the free energy functions defined in this section with the definitions of the free energy functions considered in the main text in form of the following lemma:

Lemma (Consistency of free energy function definitions).

The definitions of the variational free energy functions $\mathbb{F}^{VB}$ and F^{VB}, as well as $\mathbb{F}^{VML}$ and F^{VML} are consistent. More specifically,

(L1) *if in the definition of the variational Bayes free energy $\mathbb{F}^{VB}$(S2.36) $\tilde{B}$ and $\tilde{L}$ are continuous random vectors represented by probability density functions $q_{\tilde{B}}$ and $q_{\tilde{L}}$ with respect to Lebesgue measures μ^p_l and μ^k_l, respectively, then the definitions of $\mathbb{F}^{VB}$ and F^{VB} are equivalent, and*

(L2) *if in the definition of variational maximum likelihood free energy $\mathbb{F}^{VML}$ (S2.40) $\tilde{B}$ is a continuous random vector represented by a probability density function $q_{\tilde{B}}$ with respect to Lebesgue measure, then the definitions of $\mathbb{F}^{VML}$ and F^{VML} are equivalent.*

Proof of (L1).

Consider the definitions

$$\mathbb{F}^{VB}(Q_{\tilde{B}\otimes\tilde{L}}) := \int \ln f_{Y,B,L}(y, \cdot, \cdot)\, dQ_{\tilde{B}\otimes\tilde{L}} + \mathbb{H}(Q_{\tilde{B}\otimes\tilde{L}}) \quad \text{(S2.44)}$$

and, under the mean-field approximation,

$$F^{VB}(q(\beta)q(\lambda)) = \iint q(\beta)q(\lambda) \ln\left(\frac{p(y,\beta,\lambda)}{q(\beta)q(\lambda)}\right) d\beta d\lambda \tag{S2.45}$$

Note that the integral in (S2.44) denotes a Lebesgue integral with respect to the probability measure $Q_{\tilde{B}\otimes\tilde{L}}$, while the integral in (S2.45) denotes a (double) Riemann integral. If $\tilde{B}$ and $\tilde{L}$ are continuous random vectors with associated probability density functions $q_{\tilde{B}}$ and $q_{\tilde{L}}$, then with (S2.9) and the notational conventions of the main text, we have

$$\begin{aligned}\int \ln f_{Y,B,L}(y,\cdot,\cdot)\, dQ_{\tilde{B}\otimes\tilde{L}} &= \iint q_{\tilde{B}}(\beta)q_{\tilde{L}}(\lambda) \ln f_{Y,B,L}(y,\beta,\lambda)\, d\mu_l^p(\beta) d\mu_l^k(\lambda) \\ &= \iint q_{\tilde{B}}(\beta)q_{\tilde{L}}(\lambda) \ln f_{Y,B,L}(y,\beta,\lambda)\, d\beta d\lambda.\end{aligned} \tag{S2.46}$$

Further, because $(\tilde{B},\tilde{L})$ is a continuous random vector, (S2.26) applies for the entropy of $Q_{\tilde{B}\otimes\tilde{L}}$, and thus

$$\mathbb{H}(Q_{\tilde{B}\otimes\tilde{L}}) = h(Q_{\tilde{B}\otimes\tilde{L}}) = -\iint q_{\tilde{B}}(\beta)q_{\tilde{L}}(\lambda) \ln(q_{\tilde{B}}(\beta)q_{\tilde{L}}(\lambda))\, d\beta d\lambda. \tag{S2.47}$$

Hence, (L1) follows with the linearity of the (Riemann) integral and omission of the subscripts $\tilde{B}$ and $\tilde{L}$ in the denotation of the probability density functions $q_{\tilde{B}}$ and $q_{\tilde{L}}$.

□

Proof of (L2).

Consider the definitions

$$\mathbb{F}^{VML}(Q_{\tilde{B}},\lambda) = \int \ln f^{\lambda}_{Y,B}(y,\cdot) dQ_{\tilde{B}} + \mathbb{H}(Q_{\tilde{B}}) \tag{S2.48}$$

and

$$F^{VML}(q(\beta),\lambda) = \int q(\beta) \ln\left(\frac{p_\lambda(y,\beta)}{q(\beta)}\right) d\beta. \tag{S2.49}$$

Note that the integral in (S2.48) denotes a Lebesgue integral with respect to the probability measure $Q_{\tilde{B}}$, while the integral in (S2.49) denotes a Riemann integral. If $\tilde{B}$ is a continuous random vector with associated density function $q_{\tilde{B}}$ with respect to Lebesgue measure, then with (S2.9) and the notational conventions of the main text, we have

$$\begin{aligned}\int \ln f^{\lambda}_{Y,B}(y,\cdot) dQ_{\tilde{B}} &= \int q_{\tilde{B}}(\beta) \ln f^{\lambda}_{Y,B}(y,\beta)\, d\mu_l^p(\beta) \\ &= \int q_{\tilde{B}}(\beta) \ln p_\lambda(y,\beta)\, d\beta.\end{aligned} \tag{S2.50}$$

Further, because $\tilde{B}$ is a continuous random vector, (S2.26) applies for the entropy of $Q_{\tilde{B}}$, and thus

$$\mathbb{H}(Q_{\tilde{B}}) = h(Q_{\tilde{B}}) = -\int q_{\tilde{B}}(\beta) \ln q_{\tilde{B}}(\beta)\, d\beta. \tag{S2.51}$$

Hence, (L2) follows with the linearity of the (Riemann) integral and omission of the subscript $\tilde{B}$ in the denotation of the probability density function $q_{\tilde{B}}$.

□

Finally, we provide the key result of this section:

Theorem (Relationship of VB, VML, and ML). *Variational maximum likelihood and maximum likelihood are special cases of variational Bayes. More specifically:*

(T1) *For a constant marginal density* $f_L(\lambda) := 1$ *and the constant random vector*

$$\tilde{L}^* : \Omega \to \mathbb{R}^k, \omega \mapsto \tilde{L}^*(\omega) := \lambda^* \tag{S2.52}$$

it holds that

$$\mathbb{F}^{VB}(Q_{\tilde{B}\otimes\tilde{L}^*}) = \mathbb{F}^{VML}(Q_{\tilde{B}}, \lambda). \tag{S2.53}$$

(T2) *For a constant marginal density* $f_B(\beta) := 1$ *and the constant random vector*

$$\tilde{B}^* : \Omega \to \mathbb{R}^p, \omega \mapsto \tilde{B}^*(\omega) := \beta^* \tag{S2.54}$$

it holds that

$$\mathbb{F}^{VML}(Q_{\tilde{B}^*}, \lambda) = \mathbb{F}^{ML}(\beta^*, \lambda). \tag{S2.55}$$

Proof of (T1)

We first note that because $\tilde{B}$ and $\tilde{L}^*$ are independent random vectors and because $\tilde{L}^*$ is a constant random vector, we have with (S2.30), (S2.31), and (S2.25)

$$\mathbb{H}(Q_{\tilde{B}\otimes\tilde{L}^*}) = \mathbb{H}(Q_{\tilde{B}}) + H(Q_{\tilde{L}^*}) = \mathbb{H}(Q_{\tilde{B}}). \tag{S2.56}$$

Second, with (S2.18) we have $Q_{\tilde{L}^*} = \delta_{\lambda^*}$. Hence, with Fubini's theorem,

$$\begin{aligned}\int \ln f_{Y,B,L}\, dQ_{\tilde{B}\otimes\tilde{L}^*} &= \int \left(\int \ln f_{Y,B,L}(y,\beta,\lambda)\, d\delta_{\lambda^*} \right) dQ_{\tilde{B}} \\ &= \int \ln f_{Y,B,L}(y,\beta,\lambda^*) dQ_{\tilde{B}} \\ &= \int \ln f_{Y,B|L}(y,\beta|\lambda^*) f_L(\lambda^*) dQ_{\tilde{B}} \\ &= \int \ln f_{Y,B|L}(y,\beta|\lambda^*) dQ_{\tilde{B}},\end{aligned} \tag{S2.57}$$

where the last equality follows with $f_L(\lambda^*) = 1$. Notationally identifying the probability density function

$$f_{Y,B|L}(\cdot,\cdot|\lambda^*) : \mathbb{R}^{n+p} \to \mathbb{R}_{>0}, (y,\beta) \mapsto f_{Y,B|L}(y,\beta|\lambda^*) \tag{S2.58}$$

with the probability density function $f^{\lambda^*}_{Y,B}$ and omission of the asterisk superscript then completes the proof.

□

Proof of (T2)

We first note that because $\tilde{B}^*$ is a constant random vector, we have with (S2.25) $\mathbb{H}(Q_{\tilde{B}^*}) = 0$. Second, with (S2.18) we have $Q_{\tilde{B}^*} = \delta_{\beta^*}$. Hence,

$$\begin{aligned}\int \ln f^{\lambda}_{Y,B}(y,\cdot)\, dQ_{\tilde{B}^*} &= \int \ln f^{\lambda}_{Y,B}(y,\cdot)\, d\delta_{\beta^*} \\ &= \ln f^{\lambda}_{Y,B}(y,\beta^*) \\ &= \ln f^{\lambda}_{Y|B}(y|\beta^*)\end{aligned} \tag{S2.59}$$

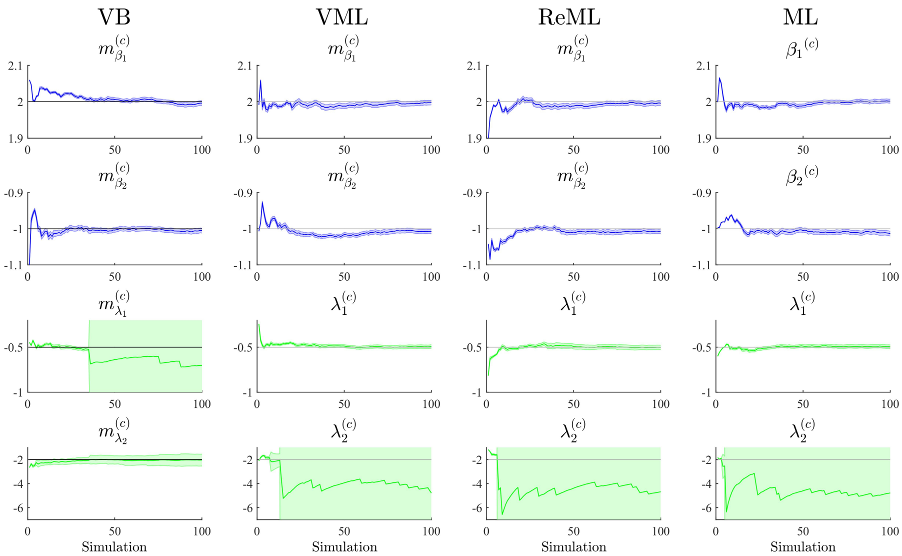

Figure S1: The panels along the figure's columns depict the cumulative averages (blue/green lines), cumulative variances (blue/green shaded areas), and true, but unknown, parameter values (grey lines) for VB, VML, ReML, and ML estimation. Parameter estimates relating to the effect sizes β are visualized in blue, parameter estimates relating to the covariance components λ are visualized in green. The panels along the figure's rows depict the parameter recovery performance for the subcomponents of the effect size parameters (row 1 and 2) and covariance component parameters (row 3 and 4), respectively. As opposed to the data shown in the main text, the covariance component parameter estimates are not corrected for outliers. For implementational details, please see *vbg_2.m*.

where the latter equality follows with $f_B(\beta^*) = 1$. Notationally identifying the probability density function

$$f_{Y|B}^{\lambda}(\cdot|\beta^*) : \mathbb{R}^n \to \mathbb{R}_{\geq 0}, y \mapsto f_{Y|B}^{\lambda}(y|\beta^*) \tag{S2.60}$$

with the probability density function $f_Y^{\beta^*,\lambda}$ and omission of the asterisk superscript then completes the proof.

□

S3 Cumulative averages without outlier removal

In Section 3.1 of the main text, we present a parameter recovery simulation for which we removed 15-20 % of outliers in the estimation of the covariance component parameters. The same results without outlier removal are depicted in Figure S1. As evident from the Figure, outliers affect all four estimation techniques, but primarily one of the covariance components. Retaining the outliers results in negative estimation bias estimates and an increase of the estimation variance estimate.

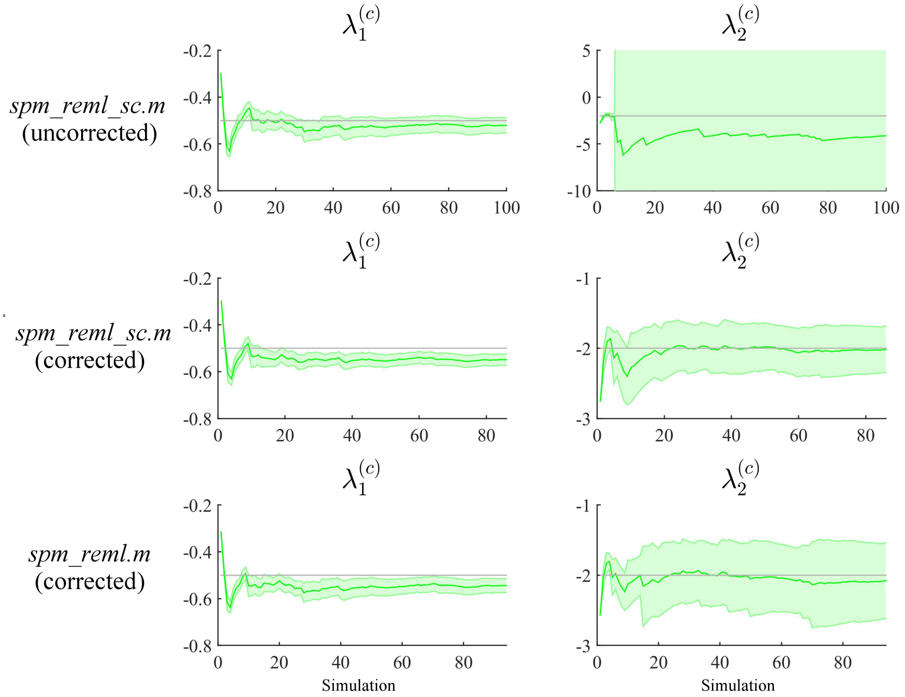

Figure S2: Parameter recovery for SPM12-based covariance component parameter estimation. The panels along the figure's columns depict the cumulative averages (gree line), cumulative variances (green shaded area), and true, but unknown, parameter values (grey) for the first and second covariance component parameters λ_1 and λ_2, respectively. The panels along the figure's rows depict these quantities for the two implementations of covariance component parameter estimation in SPM12 as indicated on the right, and without and with a correction for outliers as indicated. For implementational details, please see *vbg_2.m*.

S4 SPM12 ReML estimation

In the parameter recovery assessment of our VB, VML, ReML, and ML implementation, we found that the covariance component parameter estimation fails in a significant number of cases. To investigate whether this behaviour is specific to our implementation, we performed the same analyses using the covariance component parameter estimation functions *spm_reml_sc.m* (Version 4805) and *spm_reml.m* (Version 5223) of the SPM12 distribution. These functions perform a Fisher scoring ascent on the ReML objective function to identify maximum-a-posteriori covariance component parameter estimates, probably documented best in (Friston et al., 2002). The function *spm_reml_sc.m* uses weakly informative log normal priors to ensure the positivity of the covariance component parameter estimates, while the *spm_reml.m* function, which is called by SPM12 central *spm_spm.m* function, does not.

We visualize the results in Figure S2. The panel columns of this figure refer to the two covariance component parameter estimates and the panel rows refer to the different SPM12 functions. In the first row, we visualize

the cumulative average and variances of the respective parameter estimates based on the *spm_reml_sc.m* function without the removal of outliers. The performance for λ_1 is acceptable, but for the estimation of λ_2 outliers from approximately the 10th simulation on bias the cumulative average significantly away from the true, but unknown, parameter value and strongly amplify the cumulative variance. This is similar to the behaviour we detected in our implementation which led us to remove these outliers automatically (Grubbs, 1969). The second row of Figure S2 depicts the parameter recovery performance for *spm_reml_sc.m* after removal of appoximately 15% of outliers. This results in similar performance as in our implementation. Finally, the last row of Figure SS2 depicts the parameter recovery performance for the *spm_reml.m* function. Because *spm_reml.m* can return negative covariance components and because the SPM12 procedures assume a covariance structure of the form $V_\lambda = \sum_{i=1}^{k} \lambda_i Q_i$ and not of the form $V_\lambda = \sum_{i=1}^{k} \exp(\lambda_i) Q_i$ as in our implementation, the necessary log transformation of the returned parameter estimates here can result in undefined results. In the data shown, these undefined results have been removed, again rendering the resulting cumulative averages and variances within reasonable bounds of the true, but unknown, parameter values.

In summary, we conclude that the numerical optimization problems that we encountered for the estimation of covariance components based on our implementation of the VB, VML, ReML, and ML estimation techniques are not an uncommon phenomenon in the analysis of neuroimaging data.

S5 Model recovery free energy contributions

To understand the observed pattern of average free energies in Figure 7 in further detail, we tabulated the sum terms of each free energy function (Table S1) and visualize the average term contributions to the overall average free energy in Figure S3. We omit from visualization the first term, which is identical for all free energy functions and evaluates to T1 = -367.58. Of the remaining terms, the largest contributions are provided by T3 and T2, reflecting the residual sum of squares and the log determinant of the estimated data covariance matrix, respectively (Figure S3A). The remaining terms T4 - T17, as far as they exist for each free energy function, make smaller contributions (Figure S3B). Notably, the residual sum of squares is virtually identical over all pairings of data generating and data analysis model. This reflects the fact that the two-regressor model MA2 can readily capture the data pattern of the single-regressor model MG1 by estimating β_2 to be approximately zero. The average free energy differences for the two data analysis models in case of MG2 thus appear to be primarily accounted for by the different contributions of T2. It is likely that these differences result from the erroneous allocation of data variance under model MG2 to the covariance components of model MA1. The more subtle differences between the average free energies for MA1 and MA2 in the case of MG1 on the other hand, seem to arise from two factors: firstly, a slight overestimation of the covariance component parameters of MA2 in case of MG1, leading to a persistence of the lower average free energy values in the case of ML estimation, and secondly, from additional contributions of

T	F^{VB}	F^{VML}	F^{ReML}	F^{ML}
1	$-\frac{n}{2}\ln 2\pi$	$-\frac{n}{2}\ln 2\pi$	$-\frac{n}{2}\ln 2\pi$	$-\frac{n}{2}\ln 2\pi$
2	$-\frac{1}{2}\ln \lvert V_{m_\lambda}\rvert$	$-\frac{1}{2}\ln \lvert V_\lambda\rvert$	$-\frac{1}{2}\ln \lvert V_\lambda\rvert$	$-\frac{1}{2}\ln \lvert V_\lambda\rvert$
3	$-\frac{1}{2}(y - Xm_\beta)^T V_{m_\lambda}^{-1}(y - Xm_\beta)$	$-\frac{1}{2}(y - Xm_\beta)^T V_\lambda^{-1}(y - Xm_\beta)$	$-\frac{1}{2}(y - Xm_\beta)^T V_\lambda^{-1}(y - Xm_\beta)$	$-\frac{1}{2}(y - X\beta)^T V_\lambda^{-1}(y - X\beta)$
4	$-\frac{1}{2}\operatorname{tr}(S_\beta X^T V_{m_\lambda}^{-1} X)$	$-\frac{1}{2}\operatorname{tr}(S_\beta X^T V_\lambda^{-1} X)$	$-\frac{1}{2}\operatorname{tr}(S_\beta X^T V_\lambda^{-1} X)$	
5	$-\frac{1}{4}\operatorname{tr}(B_{m_\lambda, S_\beta, m_\lambda} S_\lambda)$			
6	$-\frac{p}{2}\ln 2\pi$	$-\frac{p}{2}\ln 2\pi$		
7	$-\frac{1}{2}\ln \lvert\Sigma_\beta\rvert$	$-\frac{1}{2}\ln \lvert\Sigma_\beta\rvert$		
8	$-\frac{1}{2}(m_\beta - \mu_\beta)^T \Sigma_\beta^{-1}(m_\beta - \mu_\beta)$	$-\frac{1}{2}(m_\beta - \mu_\beta)^T \Sigma_\beta^{-1}(m_\beta - \mu_\beta)$		
9	$-\frac{1}{2}\operatorname{tr}(\Sigma_\beta^{-1} S_\beta)$	$-\operatorname{tr}(\Sigma_\beta^{-1} S_\beta)$		
10	$-\frac{k}{2}\ln 2\pi$			
11	$-\frac{1}{2}\ln \lvert\Sigma_\lambda\rvert$			
12	$-\frac{1}{2}(m_\lambda - \mu_\lambda)^T \Sigma_\lambda^{-1}(m_\lambda - \mu_\lambda)$			
13	$-\frac{1}{2}\operatorname{tr}(\Sigma_\lambda^{-1} S_\lambda)$			
14	$+\frac{p}{2}\ln(2\pi e)$	$+\frac{p}{2}\ln(2\pi e)$	$+\frac{p}{2}\ln(2\pi e)$	
15	$+\frac{1}{2}\ln \lvert S_\beta\rvert$	$+\frac{1}{2}\ln \lvert S_\beta\rvert$	$+\frac{1}{2}\ln \lvert S_\beta\rvert$	
16	$+\frac{k}{2}\ln(2\pi e)$			
17	$+\frac{1}{2}\ln \lvert S_\lambda\rvert$			

Table S1: Free energy sum terms. Note that T1 is identical over all free energy functions, T2 is the negative log determinant of the estimated data covariance matrix, and T3 corresponds to the residual sum of squares. The remaining terms, if they exist relate to the prior and posterior uncertainties over model parameters and are commonly referred to as "model complexity" terms.

.

T4 - T16 for VB, VML, and ReML, as evident from the more negative sums (Σ) of these terms. In summary, for the current data generating and data analysis model comparison, both covariance component overestimation and the free energy model complexity terms T4 - 16 appear to contribute to the identifiability of the true, but unknown, model structure.

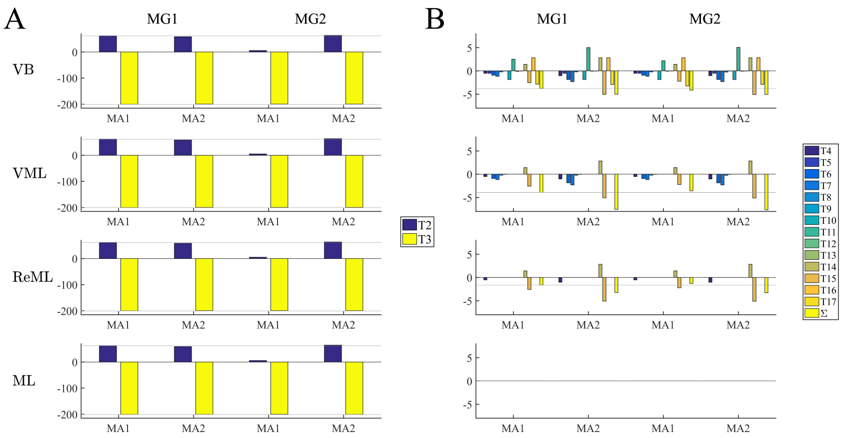

Figure S3: Average free energy term contributions. The figure depicts a decomposition of the average free energy values depicted in the main text Figure 7 according to the terms tabulated in Table S1. Panel A displays the largest contributions, afforded by terms T2 and T3, Panel B displays the remaining term contributions, and the sum (Σ) of these remaining distributions. Note the difference in scale between Panels A and B. Panel rows refer to the four estimation techniques. In each subpanel, the left two bar groups refer to data generated by MG1 analyzed with data analysis models MA1 and MA2, and the right two bar groups refer to data generated by MG2 analyzed with MA1 and MA2. For visual comparison, thin grey lines corresponding to the values obtained under the MG1/MA1 combination are included. For implementational details, please see *vbg_3.m*.

References

Bauer, H. (1991). *Wahrscheinlichkeitstheorie.(4. Aufl.) de Gruyter.*

Billingsley, P. (2012). *Probability and Measure, Anniversary Edition.* John Wiley & Sons, Inc.

Bishop, C. M. (2006). *Pattern Recognition and Machine Learning (Information Science and Statistics).* Springer-Verlag New York, Inc., Secaucus, NJ, USA.

Blei, D. M., Kucukelbir, A., and McAuliffe, J. D. (2016). Variational inference: A review for statisticians. *arXiv preprint arXiv:1601.00670.*

Coleman, T. F. and Li, Y. (1996). An interior trust region approach for nonlinear minimization subject to bounds. *SIAM Journal on optimization*, 6(2):418–445.

Cover, T. M. and Thomas, J. A. (2012). *Elements of information theory.* John Wiley & Sons.

Fristedt, B. E. and Gray, L. F. (1997). *A modern approach to probability theory.* Birkhauser.

Friston, K., Glaser, D., Henson, R. N. A., Kiebel, S., Phillips, C., and Ashburner, J. (2002). Classical and bayesian inference in neuroimaging: applications. *Neuroimage*, 16(2):484–512.

Gray, R. M. (2011). *Entropy and information theory*. Springer Science & Business Media.

Grubbs, F. E. (1969). Procedures for detecting outlying observations in samples. *Technometrics*, 11(1):1–21.

Lieb, E. H. and Loss, M. (2001). Analysis, volume 14 of graduate studies in mathematics. *American Mathematical Society, Providence, RI,*, 4.

Murphy, K. P. (2012). *Machine learning: a probabilistic perspective*. MIT press.

Nair, C., Prabhakar, B., and Shah, D. (2006). On entropy for mixtures of discrete and continuous variables. *arXiv preprint cs/0607075*.

Nocedal, J. and Wright, S. (2006). *Numerical optimization*. Springer Science & Business Media.

Petersen, K. B. and Pedersen, M. S. (2012). The matrix cookbook. Version 20121115.

Schmidt, K. D. (2011). *Mass und Wahrscheinlichkeit*. Springer.

Shao, J. (2003). *Mathematical Statistics*. Springer Texts in Statistics. Springer.
